# Association of the US Affordable Care Act With Out-of-Pocket Spending and Catastrophic Health Expenditures Among Adult Patients With Traumatic Injury

**DOI:** 10.1001/jamanetworkopen.2020.0157

**Published:** 2020-02-28

**Authors:** Charles Liu, Yusuke Tsugawa, Thomas G. Weiser, John W. Scott, David A. Spain, Melinda Maggard-Gibbons

**Affiliations:** 1 Department of Surgery, Stanford University, Stanford, California; 2 National Clinician Scholars Program, University of California, Los Angeles; 3 VA Greater Los Angeles Healthcare System, Los Angeles, California; 4 Division of General Internal Medicine and Health Services Research, David Geffen School of Medicine at UCLA, University of California, Los Angeles; 5 Department of Health Policy and Management, UCLA Fielding School of Public Health, University of California, Los Angeles; 6 Department of Surgery, University of Michigan School of Medicine, Ann Arbor; 7 Department of Surgery, David Geffen School of Medicine at UCLA, University of California, Los Angeles

## Abstract

**Importance:**

Trauma is an expensive and unpredictable source of out-of-pocket spending for American families. The Patient Protection and Affordable Care Act (ACA) sought to improve financial protection by expanding health insurance coverage, but its association with health care spending for patients with traumatic injury remains largely unknown.

**Objective:**

To evaluate the association of ACA implementation with out-of-pocket spending, premiums, and catastrophic health expenditures (CHE) among adult patients with traumatic injury.

**Design, Setting, and Participants:**

Data from a nationally representative sample of US adults aged 19 to 64 years who had a hospital stay or emergency department visit for a traumatic injury from January 2010 to December 2017 were analyzed using the Medical Expenditure Panel Survey. Multivariable generalized linear models were used to evaluate changes in spending after ACA implementation. Additionally, 4 income subgroups were evaluated based on ACA thresholds for program eligibility: lowest-income patients (earning 138% or less of the federal poverty level [FPL]), low-income patients (earning 139% to 250% of the FPL), middle-income patients (earning 251% to 400% of the FPL), and high-income patients (earning more than 400% of the FPL). Data were analyzed from February to December 2019.

**Exposures:**

Implementation of the ACA, beginning January 1, 2014.

**Main Outcomes and Measures:**

Out-of-pocket spending, premium spending, out-of-pocket plus premium spending, and likelihood of experiencing CHE, defined as out-of-pocket plus premium spending exceeding 19.5% of family income.

**Results:**

Of the 6288 included patients, 2995 (weighted percentage, 51.3%) were male, and the mean (SD) age was 41.4 (12.8) years. Implementation of the ACA was associated with 31% lower odds of CHE (adjusted odds ratio, 0.69; 95% CI, 0.54 to 0.87; *P* = .002). Changes were greatest in lowest-income patients, who experienced 30% lower out-of-pocket spending (adjusted percentage change, −30.4%; 95% CI, −46.6% to −9.4%; *P* = .01), 26% lower out-of-pocket plus premium spending (adjusted percentage change, −26.3%; 95% CI, −41.0% to −8.1%; *P* = .01), and 39% lower odds of CHE (adjusted odds ratio, 0.61; 95% CI, 0.44 to 0.84; *P* = .002). Low-income patients experienced decreased out-of-pocket spending and out-of-pocket plus premium spending but no changes in CHE, while middle-income and high-income patients experienced no significant changes in any spending outcome. In the post-ACA period, 1 in 11 of all patients with traumatic injury and 1 in 5 with the lowest incomes continued to experience CHE each year.

**Conclusions and Relevance:**

Implementation of the ACA was associated with improved financial protection for US adults with traumatic injury, especially lowest-income individuals targeted by the law’s Medicaid expansions. Despite these gains, injured patients remain at risk of financial strain.

## Key Points

**Question:**

Was the implementation of the Patient Protection and Affordable Care Act (ACA) associated with improved financial protection for US adults receiving care for traumatic injuries?

**Findings:**

In this cross-sectional study of 6288 adult patients with traumatic injury, implementation of the ACA was associated with 31% lower odds of catastrophic health expenditures. Financial protection gains were greatest in lowest-income patients targeted by Medicaid expansion, who experienced 30% lower out-of-pocket spending and 39% lower odds of catastrophic expenditures.

**Meaning:**

Implementation of the ACA was associated with improved financial protection for US adults with traumatic injury, particularly those with the lowest incomes.

## Teaser

This cross-sectional study evaluates the association of the implementation of the US Patient Protection and Affordable Care Act with out-of-pocket spending, premiums, and catastrophic health expenditures among adults with traumatic injuries.

## Introduction

Health care expenditures are a leading cause of financial hardship for US families and pose a barrier to accessing necessary medical care.^1,2,3^ In 2015, 1 in 4 US adults aged 18 to 64 years reported their household had problems paying medical bills, and 60% of these adults reported a household member had delayed needed care because of cost.^4^ This burden results from both lack of insurance and underinsurance; 45% of uninsured adults report difficulty paying bills, as do 25% of insured adults.^5^ In particular, traumatic injury is highly unpredictable and expensive and disproportionately affects socioeconomically disadvantaged populations. Recent research has shown that more than 70% of uninsured patients who experience traumatic injury in the United States are at risk of incurring catastrophic out-of-pocket expenditures.^6^ In contrast to other types of illness, trauma is less likely to be influenced by improved access to primary and preventive care and remains a substantial source of financial risk to patients despite ongoing efforts to improve access.

The Patient Protection and Affordable Care Act (ACA) sought to protect US families from high and rising health care expenditures by expanding health insurance coverage and reducing underinsurance through various avenues.^7,8,9^ In many states, individuals earning up to 138% of the federal poverty level (FPL; $16 643 per year for individuals and $33 948 per year for families of 4) became eligible for Medicaid, leading to 13 million newly eligible Americans enrolling in coverage.^10,11^ Additionally, through the ACA insurance Marketplaces, individuals earning up to 400% of the FPL ($48 240 per year for individuals and $98 400 per year for families of 4) were eligible to receive premium subsidies to reduce the cost of purchasing private insurance and those earning up to 250% of the FPL ($30 150 per year for individuals and $61 500 per year for families of 4) were also eligible for reduced out-of-pocket costs through cost-sharing subsidies provided to insurers.^12^ To date, 10 million Americans have purchased coverage through the ACA Marketplaces. The implementation of the ACA has been shown to be associated with decreased barriers to medical care as well as reduced out-of-pocket spending, both among individuals affected by the Medicaid expansions^13,14,15^ and those eligible for Marketplace subsidies.^16,17^ Recent research has also demonstrated an association between Medicaid expansion and reduced catastrophic expenditures among injured patients in Washington state.^18^ However, to our knowledge, no studies have evaluated the association of the ACA with financial risk protection for patients with traumatic injury nationally.

In this context, we examined the association of the implementation of the ACA with out-of-pocket and premium spending among adults with traumatic injuries. We hypothesized that ACA implementation was associated with improved insurance coverage, lower out-of-pocket spending, and improved protection from catastrophic expenditures among these patients.

## Methods

### Data and Study Population

We analyzed the 2010 to 2017 Medical Expenditure Panel Survey (MEPS), a nationally representative survey of health care use and expenditures by the US civilian noninstitutionalized population.^19^ The Medical Expenditure Panel Survey collects data on out-of-pocket spending, premiums, income, and demographic characteristics from approximately 15 000 households per year, interviewing each household 5 times over a 2-year period. Information from respondents is supplemented with data from hospitals, medical offices, and pharmacies to produce highly valid estimates for expenditures.

Our study population included US adults aged 19 to 64 years who had an inpatient hospital stay or emergency department (ED) visit for trauma between January 1, 2010, and December 31, 2017. Trauma-related encounters were identified by the presence of either an *International Classification of Diseases, Ninth Revision, Clinical Modification *(*ICD*-*9*-*CM*; 2010 to 2015) or an *ICD*-*10*-*CM *(2016 and 2017) diagnosis code for trauma (eTables 1 and 2 in the Supplement) or the injury indicator variable, defined by MEPS as “physical problems because of some sort of external trauma to the body such as a fall or being in an auto accident.”^20^ As the major provisions of the ACA took effect on January 1, 2014, we defined the pre-ACA and post-ACA periods as January 2010 to December 2013 and January 2014 to December 2017, respectively.

In addition to the full sample, patients were also stratified into 4 income subgroups for analysis: (1) lowest-income patients (earning 138% or less of the FPL) eligible to gain Medicaid coverage through the ACA’s Medicaid expansions, (2) low-income patients (earning 139% to 250% of the FPL) eligible for cost-sharing and premium subsidies on the ACA Marketplaces, (3) middle-income patients (earning 251% to 400% of the FPL) eligible for only premium subsidies, and (4) high-income patients (earning more than 400% of the FPL) ineligible for subsidies. Ethical approval was obtained from the University of California, Los Angeles Institutional Review Board, including a waiver of informed consent for this analysis of deidentified data. This study followed the Strengthening the Reporting of Observational Studies in Epidemiology (STROBE) reporting guideline for cross-sectional studies.^21^

### Definitions of Health Care Spending

All expenditures were adjusted for inflation using the Personal Health Care Index^22^ and incomes using the Consumer Price Index.^23,24,25^ Our primary outcome was the individual’s out-of-pocket health care spending for the calendar year during which an inpatient stay or ED visit for trauma occurred. We included spending from all types of health care encounters, including inpatient stays, outpatient and ED visits, prescription drugs, and home health visits, given that care for trauma and its sequelae spans a broad range of settings and that postacute care is a key driver of variation in spending following trauma.^26,27,28^ We also evaluated premium and out-of-pocket plus premium spending, which were summed over the patient’s family, as MEPS does not attribute premiums to individual family members.

In addition to analyzing spending as a continuous variable, we also studied the percentage of patients meeting the threshold for catastrophic health expenditures (CHE). To do so, we summed out-of-pocket and premium spending for all members of the patient’s family and divided by their combined income. We used the Current Population Survey (CPS) definition of family, which includes individuals living together and related by birth, marriage, or adoption.^29,30^ We defined CHE as out-of-pocket plus premium spending exceeding 19.5% of family income. This definition has been used in prior research^17^ and is derived from the sum of (1) a widely used threshold for catastrophic out-of-pocket spending as that exceeding 10% of family income^31,32,33^ and (2) a 9.5% income threshold for high-burden premiums, based on an ACA provision that allows individuals whose employer-based insurance premiums exceed 9.5% of income to instead purchase a plan on the ACA Marketplaces.^34^

### Statistical Analysis

Changes in sociodemographic characteristics, health status, and insurance status from before to after ACA implementation were assessed using *t* tests for continuous variables and χ^2^ tests for categorical variables. We examined the association between ACA implementation and financial risk protection by fitting multivariable generalized linear models with a log-link function and gamma distribution for the continuous outcomes and a multivariable logistic regression model for the binary outcome (ie, CHE). Analyses were carried out for the full sample as well as each of the 4 income subgroups. We adjusted for a set of potential confounders identified a priori through a conceptual approach, which included age, sex, race/ethnicity, marital status, fair/poor self-reported health, census region, unemployment, and family size. Additionally, we adjusted for changes in injury types by including dummy variables for 6 groups of diagnosis codes (eTable 3 in the Supplement). Survey weights, strata, and clusters provided by MEPS were used to account for the complex survey design and to produce nationally representative estimates from our sample, and cluster-robust standard errors were used to account for the clustering of observations within each primary sampling unit (a group of neighboring counties). We used 2-tailed tests and a significance threshold of *P* < .05. All final analyses were conducted using Stata/SE version 16.0 (StataCorp). Data were analyzed from February to December 2019.

### Sensitivity Analyses

We conducted 6 sensitivity analyses. First, only patients identified using the injury flag in MEPS were included to avoid possible inconsistencies introduced by the switch from *ICD*-*9*-*CM *to *ICD*-*10*-*CM*. Second, young adults aged 19 to 25 years who may have gained insurance in 2010 under the ACA’s Dependent Coverage Provision, which allows them to remain on their parents’ health insurance, were excluded. Third, only patients with an inpatient hospital stay for trauma were included. Fourth, the MEPS definition instead of the CPS definition of family was used, which includes nonmarried partners, foster children, and in-laws. Fifth, linear regression was used instead of logistic regression to evaluate CHE. Sixth, a 2-part model was used to evaluate changes in continuous spending outcomes.

## Results

Our analysis included 6288 patients with traumatic injury over the 8-year study period. A total of 2995 patients (weighted percentage, 51.3%) were male, and the mean (SD) age was 41.4 (12.8) years. From the pre-ACA to the post-ACA period, there was a slight increase in nonwhite race overall and in the middle-income subgroup and a slight increase in age overall and in the lowest-income subgroup, with no significant change in other demographic characteristics (Table 1). Mean family income increased by approximately 10%, adjusting for inflation. Notably, the uninsured rate decreased from 22.2% to 15.1% in the full cohort (*P* < .001) and from 34.6% to 23.5% in the lowest-income subgroup (*P* < .001), and significant decreases were seen in the low-income and middle-income subgroups as well. At the same time, Medicaid coverage increased from 16.6% to 24.7% in the full cohort (*P* < .001) and from 42.7% to 55.4% in the lowest-income subgroup (*P* < .001), and significant increases in Medicaid coverage were also seen in the 3 higher-income subgroups. Finally, coverage purchased on the ACA Marketplaces increased from 0 to 3.6% of the study population in the post-ACA period.

**Table 1.** Characteristics of US Adults Aged 19 to 64 Years With an Inpatient Stay or Emergency Department Visit for Trauma Between 2010 and 2017 by Income Category

Characteristic	Weighted %									
	All Patients		Income Subgroup^a^							
			Lowest		Low		Middle		High	
	Pre-ACA Period	Post-ACA Period	Pre-ACA Period	Post-ACA Period	Pre-ACA Period	Post-ACA Period	Pre-ACA Period	Post-ACA Period	Pre-ACA Period	Post-ACA Period
Total, No.	3254	3034	1193	1113	767	654	591	559	703	708
Population, weighted No.	32 090 478	28 753 098	9 182 104	8 215 787	6 956 035	5 473 242	6 554 211	5 319 041	9 398 128	9 745 029
Age, mean (SD), y	40.9 (12.6)	41.9 (13.1)^b^	38.6 (14.3)	40.5 (14.9)^b^	39.1 (12.4)	39.5 (13.6)	40.0 (11.6)	41.4 (12.5)	45.0 (10.7)	44.9 (10.8)
Female	48.4	49.1	55.6	57.2	49.1	49.9	43.6	41.7	44.3	45.9
Race/ethnicity										
Non-Hispanic white	68.1	63.1^b^	58.1	55.5	65.0	59.5	73.7	60.7^b^	76.3	72.9
Hispanic	11.6	14.1^b^	14.1	15.4	14.8	17.5	10.5	16.0^b^	7.5	10.2
Non-Hispanic black	14.9	15.7^b^	20.8	21.3	16.6	16.6	12.6	17.1^b^	9.4	9.6
Asian	2.0	2.5^b^	1.8	1.1	1.7	2.2	1.1	2.7^b^	3.1	3.6
Other/multiple	3.4	4.6^b^	5.1	6.7	1.9	4.3	2.2	3.5^b^	3.7	3.7
Marital status										
Married	43.0	43.4	22.2	22.7	37.9	40.3	47.4	48.6	64.1	59.9
Divorced, separated, or widowed	21.7	22.6	33.5	33.5	22.8	21.7	16.8	20.0	12.9	15.4
Never married	35.2	34.0	44.3	43.9	39.3	38.0	35.8	31.4	23.0	24.7
Census region										
Northeast	19.0	19.5	14.1	16.4	18.7	14.3	18.1	19.1	24.7	25.0
Midwest	24.6	24.5	27.5	25.1	21.5	25.4	25.7	25.2	23.4	23.0
South	35.3	34.8	37.2	39.2	39.1	39.1	36.4	37.9	29.8	29.9
West	21.1	20.2	21.2	19.2	20.7	20.8	19.8	17.7	22.2	22.1
Employed	72.6	73.8	48.6	50.6	74.4	74.8	84.4	83.8	86.6	87.4
Family income, mean SD, $^c^	61 923 (55 655)	68 365 (67 747)^b^	12 830 (10 529)	12 805 (10 193)	37 087 (14 845)	36 745 (14 572)	62 589 (19 936)	60 985 (21 145)	127 805 (52 603)	136 995 (63 610)
Family size, mean (SD)	2.7 (1.4)	2.6 (1.4)	2.6 (1.8)	2.4 (1.8)	2.8 (1.6)	2.9 (1.7)	2.8 (1.4)	2.7 (1.4)	2.6 (1.0)	2.6 (1.0)
Self-reported health										
Excellent/very good/good	75.8	75.6	60.6	61.1	75.5	75.7	79.5	76.8	88.3	87.1
Fair/poor	24.1	23.9	39.1	37.7	24.2	24.2	20.5	22.7	11.8	12.8
Unknown	0.2	0.5	0.3	1.2	0.3	0.2	0	0.5	0	0.1
Insurance coverage^d^										
Private	56.6	55.7	17.1	14.3	51.7	44.1^b^	72.6	72.5	87.7	87.8
Marketplace	NA	3.6	NA	3.2	NA	5.4	NA	3.6	NA	3.1
Medicaid	16.6	24.7^b^	42.7	55.4^b^	14.6	28.7^b^	4.4	11.7^b^	1.2	3.9^b^
Uninsured	22.2	15.1^b^	34.6	23.5^b^	28.8	21.6^b^	18.1	11.5^b^	8.0	6.3

Abbreviations: ACA, Patient Protection and Affordable Care Act; NA, not applicable.

^a^ Income subgroups were based on ACA thresholds for program eligibility and included lowest-income patients (earning 138% or less of the federal poverty level), low-income patients (earning 139% to 250% of the federal poverty level), middle-income patients (earning 251% to 400% of the federal poverty level), and high-income patients (earning more than 400% of the federal poverty level).

^b^ *P* < .05 for difference from pre-ACA period in same income group using *t* test for continuous variables and χ^2^ test for categorical variables.

^c^ Income provided in 2017 US dollars.

^d^ Reflects insurance coverage sources in December of each study year. Individuals may report more than 1 source of insurance coverage, so percentages may sum to greater than 100%. Private includes commercial insurance coverage obtained through an employer, on the ACA marketplaces, or through other sources.

### Out-of-Pocket and Premium Spending

In the full sample, we did not observe a significant association between ACA implementation and changes in out-of-pocket spending, premium spending, or out-of-pocket plus premium spending (Table 2). However, among the lowest-income patients, ACA implementation was associated with a 30.4% decrease (95% CI, −46.6% to −9.4%; *P* = .01) in out-of-pocket spending and a 26.3% decrease (95% CI, −41.0% to −8.1%; *P* = .01) in out-of-pocket plus premium spending. Similarly, in the low-income subgroup, ACA implementation was associated with a 21.4% decrease (95% CI, −34.5% to −5.7%; *P* = .01) in out-of-pocket spending and a 17.6% decrease (95% CI, −30.2% to −2.8%; *P* = .02) in out-of-pocket plus premium spending. No significant change in spending was seen in middle-income and high-income patients.

**Table 2.** Out-of-Pocket and Premium Spending Among US Adults With Traumatic Injury Before and After Implementation of the ACA^a^

Income Category^b^	Mean, $^c^		Adjusted % Change (95% CI)	*P* Value
	Spending in the Pre-ACA Period	Estimated Change in Spending		
Out-of-pocket spending				
All patients	1105	−47	−4.3 (−15.5 to 8.5)	.49
Lowest-income patients	858	−287	−30.4 (−46.6 to −9.4)	.005
Low-income patients	972	−219	−21.4 (−34.5 to −5.7)	.009
Middle-income patients	1346	−102	−7.6 (−28.3 to 19.2)	.54
High-income patients	1278	175	14.1 (−2.1 to 33.0)	.09
Premium spending				
All patients	2109	173	8.0 (−2.8 to 20.0)	.15
Lowest-income patients	437	6	1.2 (−32.0 to 50.6)	.95
Low-income patients	1758	−390	−20.4 (−37.2 to 1.0)	.06
Middle-income patients	2479	−13	−0.5 (−16.6 to 18.7)	.95
High-income patients	3743	421	11.4 (−1.8 to 26.4)	.09
Out-of-pocket and premium spending				
All patients	3953	−45	−1.1 (−8.7 to 7.0)	.78
Lowest-income patients	1624	−451	−26.3 (−41.0 to −8.1)	.006
Low-income patients	3332	−596	−17.6 (−30.2 to −2.8)	.02
Middle-income patients	4732	−157	−3.4 (−17.1 to 12.6)	.66
High-income patients	6146	473	7.7 (−1.7 to 18.1)	.11

Abbreviation: ACA, Patient Protection and Affordable Care Act.

^a^ Out-of-pocket spending is summed over each individual; premium and combined out-of-pocket and premium spending are summed over each family. Regression analyses were performed using the individual as the unit of analysis.

^b^ Income subgroups were based on ACA thresholds for program eligibility and included lowest-income patients (earning 138% or less of the federal poverty level), low-income patients (earning 139% to 250% of the federal poverty level), middle-income patients (earning 251% to 400% of the federal poverty level), and high-income patients (earning more than 400% of the federal poverty level).

^c^ Income provided in 2017 US dollars.

### Likelihood of CHE

In the full sample, ACA implementation was associated with a 31% decrease in odds of CHE (adjusted odds ratio, 0.69; 95% CI, 0.54 to 0.87; *P* = .002) (Table 3). Individuals in the lowest-income subgroup experienced a 39% decrease in odds of CHE (adjusted odds ratio, 0.61; 95% CI, 0.44 to 0.84; *P* = .002). No significant change in the likelihood of CHE was seen in the 3 higher-income groups. Rates of CHE remained high after ACA implementation, at 8.9% overall and 19.9% in the lowest-income subgroup (Figure).

**Table 3.** Likelihood of CHE Among US Adults With Traumatic Injury Before and After Implementation of the ACA^a^

Income Category^b^	Likelihood of CHE, %		Adjusted Odds Ratio (95% CI)	*P* Value
	Pre-ACA Period	Post-ACA Period		
All patients	12.2	8.9	0.69 (0.54-0.87)	.002
Lowest-income patients	28.9	19.9	0.61 (0.44-0.84)	.002
Low-income patients	10.7	8.6	0.72 (0.43-1.20)	.21
Middle-income patients	5.0	5.5	1.04 (0.49-2.22)	.91
High-income patients	1.9	1.7	0.83 (0.35-2.01)	.68

Abbreviations: ACA, Patient Protection and Affordable Care Act; CHE, catastrophic health expenditures.

^a^ Determination of meeting catastrophic expenditure thresholds is conducted using family combined spending and income; regression analyses were performed using the individual as the unit of analysis.

^b^ Income subgroups were based on ACA thresholds for program eligibility and included lowest-income patients (earning 138% or less of the federal poverty level), low-income patients (earning 139% to 250% of the federal poverty level), middle-income patients (earning 251% to 400% of the federal poverty level), and high-income patients (earning more than 400% of the federal poverty level).

**Figure.**
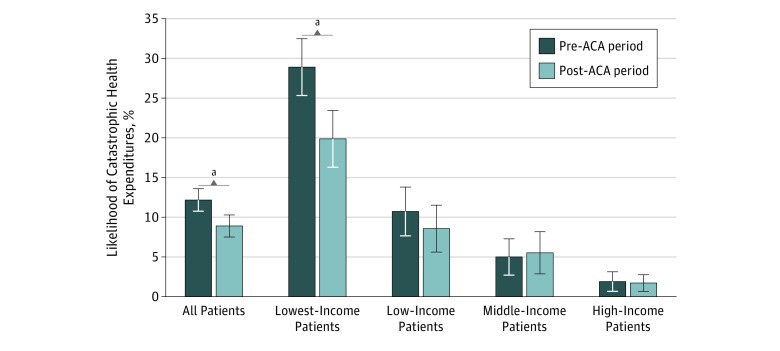
Likelihood of Catastrophic Health Expenditures Among US Adults With Traumatic Injury Before and After Implementation of the Affordable Care Act (ACA) The study sample included 6288 adults with traumatic injury, representing approximately 61 million US adults after accounting for survey weights. Univariate *P* values for the difference in CHE likelihood before and after implementation of the ACA for all patients and the 4 income subgroups were *P* = .003, *P* = .002, *P* = .35, *P* = .76, and *P* = .83, respectively. Error bars indicate 95% CIs. ^a^*P* < .01.

### Sensitivity Analyses

Sensitivity analyses 1, 2, 4, and 6 did not qualitatively affect the results of our analyses of out-of-pocket and premium spending, with the exception that including only patients identified by the injury flag resulted in the decrease in out-of-pocket plus premium spending in the low-income subgroup becoming nonsignificant (adjusted percentage change, −13.9%; 95% CI, −27.0% to 1.4%; *P* = .07) (eTables 4, 5, 6, 7, 8, and 9 in the Supplement). Conversely, when excluding patients aged 19 to 25 years, we observed a statistically significant decrease in premium spending in the low-income subgroup. Including only patients with an inpatient stay resulted in the spending decreases in the lowest-income group becoming nonsignificant, likely because of decreased sample size.

In our analysis of CHE, sensitivity analyses 1, 2, 4, and 5 did not qualitatively affect our results (eTable 10 in the Supplement). Including only patients with an inpatient stay resulted in the observed decreases in CHE odds becoming nonsignificant, likely because of decreased sample size.

## Discussion

Using nationally representative data, we found that implementation of the ACA in January 2014 was associated with decreased out-of-pocket spending among low-income US adults with traumatic injuries who were eligible for the policy’s Medicaid expansions and Marketplace subsidies. Implementation of the ACA was also associated with decreased odds of experiencing CHE, especially for the lowest-income patients earning 138% or less of the FPL. The decreases in spending were substantial in magnitude, ranging from an 18% decrease in out-of-pocket plus premium spending among low-income patients to a 30% decrease in out-of-pocket spending among lowest-income patients. Notably, these improvements in financial protection were not seen in middle-income adults eligible only for premium subsidies or high-income individuals ineligible for subsidies. Furthermore, our findings illustrate that strikingly high rates of CHE persist among patients with traumatic injury in the post-ACA period.

Compared with previous studies of medical expenditures in all adults, we found higher rates of CHE among patients with traumatic injuries. In the pre-ACA period, 29% of lowest-income patients experienced CHE each year compared with 16% of all nonelderly US adults with the same income.^17^ Among lowest-income patients who were admitted to the hospital with trauma, the likelihood of CHE before implementation of the ACA was higher still, at 37%. Lowest-income patients also experienced larger gains in financial protection than all nonelderly US adults with the same income, seeing a 39% decrease in odds of CHE vs a 16% decrease in all adults.^17^

To our knowledge, our study is the first to examine catastrophic expenditures among US patients with traumatic injury using out-of-pocket spending reported directly by households. However, true payments made may underestimate the financial burden imposed by trauma, since low-income patients are often unable to pay medical bills and may face wage garnishment, debt, and collection practices (such as home liens) that are not captured in MEPS.^2,35,36^ Previous studies using modeled out-of-pocket payments based on hospital charges have estimated the risk of CHE in patients admitted for trauma before implementation of the ACA at 26% for the privately insured and 70% for the uninsured.^6,18^ The true rate of CHE following traumatic injury may fall between these estimates and ours (12% in all patients with traumatic injury and 29% in the lowest-income patients) due to the unmeasured forms of financial hardship described above.

Several possible reasons exist for the association of ACA implementation with improved financial protection among patients with traumatic injury. First, the uninsured rate decreased in our full cohort as well as the 3 lower-income subgroups, while Medicaid coverage increased overall and in all subgroups. Since Medicaid generally has little to no cost sharing or premiums,^37,38^ these enrollment increases likely contributed to decreases in CHE. While the ACA only directly funded Medicaid expansion to individuals earning up to 138% of the FPL, we observed increased Medicaid coverage in higher-income groups as well. This may be because of the spillover effects of state and federal investment in Medicaid and ACA Marketplace enrollment,^39^ as individuals earning more than 138% of the FPL may be Medicaid eligible because of membership in a qualified group (eg, disabled people, low-income individuals with children) but unaware of their eligibility.

Second, in addition to increased Medicaid coverage, approximately 4% of patients in our study reported ACA Marketplace insurance coverage after ACA implementation, which is required by law to cover hospital and ED care, including for trauma, and to cap annual out-of-pocket spending.^7,9^ If these patients were previously uninsured or underinsured, gaining Marketplace coverage would thus lead to reduced out-of-pocket costs and catastrophic expenditures. Third, even for patients with non-Medicaid and non-Marketplace coverage, the law’s establishment of essential health benefits and its ban on price discrimination for preexisting conditions likely resulted in improved financial protection.^9,40^ Finally, improved access to primary and preventive care through the ACA^8^ may have led to better management of comorbidities, potentially leading to fewer complications when patients present with traumatic injuries.

We did not observe decreases in CHE among low-income and middle-income patients despite their eligibility for ACA Marketplace subsidies, which may be because of several reasons. First, these groups had higher rates of insurance coverage before implementation of the ACA than the lowest-income subgroup, so gains in insurance coverage following ACA implementation were correspondingly smaller. Second, Marketplace (and private non-Marketplace) insurance plans have higher copayments and deductibles than Medicaid, and states are permitted to charge premiums to Medicaid beneficiaries earning more than 150% of the FPL,^37^ so the types of coverage gained by these subgroups did not lower spending as much. Third, because of lower ACA subsidies and higher income, middle-income individuals in particular may have tended to purchase less generous Marketplace plans, exposing them to higher out-of-pocket costs in the event of trauma. For example, during the 2018 Marketplace open enrollment period, individuals earning 100% to 250% of the FPL selected silver-tier or gold-tier plans over bronze-tier plans 82% to 18%, while those earning 251% to 400% of the FPL selected bronze-tier plans over silver-tier or gold-tier plans 51% to 49%.^41^ (Platinum and catastrophic plan enrollment was not released in these data.) Fourth, low-income and middle-income patients in our study had employer-sponsored insurance more often (40% and 66%, respectively) than the lowest-income patients (9%). Underinsurance has grown substantially among Americans with employer-sponsored insurance over the past 15 years,^42^ possibly blunting gains in financial protection from ACA-related increases in Medicaid and Marketplace coverage.

Finally, we found that even after ACA implementation, nearly 1 in 11 of all US patients with trauma and 1 in 5 with incomes of 138% or less of the FPL continued to experience catastrophic health care spending. Among patients experiencing CHE in the post-ACA period, one-fifth remained residually uninsured, and nearly three-fourths were insured (half with private insurance and one-quarter with Medicaid) but continued to experience CHE, indicating that they are underinsured (eTable 11 in the Supplement). This may be because of variable implementation of the ACA in different states, lack of awareness of program eligibility, or plan-specific factors, such as out-of-network billing. There remains a critical need for policy solutions to address this crisis of affordability among low-income patients struggling with the dual misfortunes of traumatic injury and high-burden health care costs.

### Limitations

Our study has several limitations. First, as previously mentioned, since MEPS does not quantify unpaid bills or medical debt, we are unable to assess the association of the ACA with these forms of financial hardship. Second, diagnoses in MEPS are self-reported and patients with traumatic injury may thus be undercaptured, and the definition of injury used by MEPS is broad. However, both of these features are likely randomly distributed in all income groups and time periods and would thus not bias our regression estimates. Third, our sample has lower injury severity than populations in most trauma registries, which generally enroll only inpatients. As a result, our study may underestimate true rates of CHE among US patients with trauma. We attempted to address this through our sensitivity analysis that includes only patients with a hospital stay for trauma (eTables 4, 5, 6, and 10 in the Supplement). Fourth, while we adjusted for census region in our models, some residual confounding owing to geographic variation in health care costs is possible, for example, due to patient redistribution within census regions during the study period.

## Conclusions

Using nationally representative data, we found that low-income US adults with traumatic injuries experienced significant decreases in out-of-pocket spending and rates of catastrophic expenditures following implementation of the ACA. As the future of the ACA remains hotly debated, with 14 states having not adopted Medicaid expansion as of November 2019,^43^ cost-sharing subsidy payments to insurers halted in October 2017,^44^ and the individual mandate eliminated in January 2019,^45^ our findings provide evidence that the ACA was associated with significant decreases in the risk of financial catastrophe caused by trauma.

## Notes

Supplement. **eTable 1.** Clinical Classification Software (CCS) Codes Used to Identify Trauma Patients From 2010 to 2015 **eTable 2.** *ICD*-*10*-*CM* Code Categories Used to Identify Trauma Patients in 2016 and 2017 **eTable 3.** Diagnostic Groupings Used to Control for Changes in Injury Types Between Pre- and Post-ACA Periods **eTable 4.** Sensitivity Analyses Using Alternate Regression Specifications for Out-of-Pocket Spending **eTable 5.** Sensitivity Analyses Using Alternate Regression Specifications for Premium Spending **eTable 6.** Sensitivity Analyses Using Alternate Regression Specifications for Out-of-Pocket and Premium Spending **eTable 7.** Two-Part Model Results for Out-of-Pocket Spending (Sensitivity Analysis 6) **eTable 8.** Two-Part Model Results for Premium Spending (Sensitivity Analysis 6) **eTable 9.** Two-Part Model Results for Out-of-Pocket and Premium Spending (Sensitivity Analysis 6) **eTable 10.** Sensitivity Analyses Using Alternate Regression Specifications for Catastrophic Health Expenditures **eTable 11.** Insurance Coverage Among Trauma Patients Aged 19 to 64 Years Who Experienced Catastrophic Health Expenditures in the Post-ACA Period (2014 to 2017) **eReferences. **
