# Supplementary material for: Association of the US Affordable Care Act With Out-of-Pocket Spending and Catastrophic Health Expenditures Among Adult Patients With Traumatic Injury
Source: JAMA Netw Open. 2020 Feb 28;3(2):e200157. doi: 10.1001/jamanetworkopen.2020.0157 (PMC7049078; doi:10.1001/jamanetworkopen.2020.0157)
Supplement: Supplement. — eTable 1. Clinical Classification Software (CCS) Codes Used to Identify Trauma Patients From 2010 to 2015 eTable 2. ICD-10-CM Code Categories Used to Identify Trauma Patients in 2016 and 2017 eTable 3. Diagnostic Groupings Used to Control for Changes in Injury Types Between Pre- and Post-ACA Periods eTable 4. Sensitivity Analyses Using Alternate Regression Specifications for Out-of-Pocket Spending eTable 5. Sensitivity Analyses Using Alternate Regression Specifications for Premium Spending eTable 6. Sensitivity Analyses Using Alternate Regression Specifications for Out-of-Pocket and Premium Spending eTable 7. Two-Part Model Results for Out-of-Pocket Spending (Sensitivity Analysis 6) eTable 8. Two-Part Model Results for Premium Spending (Sensitivity Analysis 6) eTable 9. Two-Part Model Results for Out-of-Pocket and Premium Spending (Sensitivity Analysis 6) eTable 10. Sensitivity Analyses Using Alternate Regression Specifications for Catastrophic Health Expenditures eTable 11. Insurance Coverage Among Trauma Patients Aged 19 to 64 Years Who Experienced Catastrophic Health Expenditures in the Post-ACA Period (2014 to 2017) eReferences. [file jamanetwopen-3-e200157-s001.pdf]

Supplementary Online Content

Liu C, Tsugawa Y, Weiser TG, Scott JW, Spain DA, Maggard-Gibbons M. Association of the US Affordable Care Act with out-of-pocket spending and catastrophic health expenditures among adult patients with traumatic injury. *JAMA Netw Open*. 2020;3(2):e200157. doi:10.1001/jamanetworkopen.2020.0157

eTable 1. Clinical Classification Software (CCS) Codes Used to Identify Trauma Patients From 2010 to 2015
eTable 2. *ICD-10-CM* Code Categories Used to Identify Trauma Patients in 2016 and 2017
eTable 3. Diagnostic Groupings Used to Control for Changes in Injury Types Between Pre- and Post-ACA Periods
eTable 4. Sensitivity Analyses Using Alternate Regression Specifications for Out-of-Pocket Spending
eTable 5. Sensitivity Analyses Using Alternate Regression Specifications for Premium Spending
eTable 6. Sensitivity Analyses Using Alternate Regression Specifications for Out-of-Pocket and Premium Spending
eTable 7. Two-Part Model Results for Out-of-Pocket Spending (Sensitivity Analysis 6)
eTable 8. Two-Part Model Results for Premium Spending (Sensitivity Analysis 6)
eTable 9. Two-Part Model Results for Out-of-Pocket and Premium Spending (Sensitivity Analysis 6)
eTable 10. Sensitivity Analyses Using Alternate Regression Specifications for Catastrophic Health Expenditures
eTable 11. Insurance Coverage Among Trauma Patients Aged 19 to 64 Years Who Experienced Catastrophic Health Expenditures in the Post-ACA Period (2014 to 2017)
eReferences.

This supplementary material has been provided by the authors to give readers additional information about their work.

© 2020 Liu et al. *JAMA Network Open.*

eTable 1. Clinical Classification Software (CCS) Codes Used to Identify Trauma Patients From 2010 to 2015*

CCS Code	CCS Code Description	Representative ICD-9-CM Code(s)	Representative ICD-9-CM Code(s) Description
225	Joint disorders and dislocations; trauma-related	832.01<br>836.0	Anterior dislocation of elbow, closed<br>Tear of medial cartilage or meniscus of knee, current
226	Fracture of neck of femur (hip)	820.8	Fracture of unspecified part of neck of femur, closed
227	Spinal cord injury	806.02<br>952.00	Fracture of vertebral column, C1-C4 level w anterior cord syndrome, closed<br>Spinal cord injury without evidence of spinal bone injury, C1-C4 level with unspecified spinal cord injury
228	Skull and face fractures	800.06<br>802.4	Fracture of vault of skull, closed without mention of intracranial injury, with loss of consciousness of unspecified duration<br>Fracture of malar and maxillary bones, closed
229	Fracture of upper limb	810.02<br>814.01	Fracture of shaft of clavicle, closed<br>Fracture of scaphoid of wrist, closed
230	Fracture of lower limb	823.22<br>824.6	Fracture of tibia with fibula, shaft, closed<br>Trimalleolar ankle fracture, closed
231	Other fractures	807.09<br>808.0	Fracture of multiple ribs, unspecified, closed<br>Fracture of acetabulum, closed
232	Sprains and strains	844.1<br>845.00	Sprain of medial collateral ligament of knee<br>Sprain of ankle, unspecified site
233	Intracranial injury	850.11<br>852.21	Concussion with loss of consciousness of 30 minutes or less<br>Subdural hemorrhage following injury without mention of open intracranial wound, with no loss of consciousness
234	Crushing injury or internal injury	860.0<br>865.01	Traumatic pneumothorax without mention of open wound into thorax<br>Splenic hematoma without rupture of capsule, without mention of open wound into cavity
235	Open wounds of head; neck; and trunk	876.0<br>877.1	Open wound of back, without mention of complication<br>Open wound of buttock, complicated
236	Open wounds of extremities	882.2<br>895.0	Open wound of hand except finger(s) alone, with tendon involvement<br>Traumatic amputation of toe(s), without mention of complication
239	Superficial injury; contusion	911.0<br>916.8	Abrasion or friction burn of trunk, without mention of infection<br>Other and unspecified superficial injury of hip, thigh, leg, and ankle without mention of infection
240	Burns	942.23	Burn of abdominal wall, blisters, epidermal loss [second degree]
244	Other injuries and conditions due to external causes	958.1<br>958.92	Fat embolism<br>Traumatic compartment syndrome of lower extremity

© 2020 Liu et al. *JAMA Network Open.*

* CCS codes were used to identify trauma patients in 2010-2015 and ICD-10-CM codes were used to identify trauma patients in 2016-2017. CCS codes represent clinical groupings of ICD-9-CM diagnosis codes and were developed by the Healthcare Cost and Utilization Project for research and administrative purposes.[1]

© 2020 Liu et al. *JAMA Network Open.*

eTable 2. *ICD-10-CM* Code Categories Used to Identify Trauma Patients in 2016 and 2017*

ICD-10-CM Code Category	ICD-10-CM Code Category Description	Representative ICD-10-CM Code(s)	Representative ICD-9-CM Code(s) Description
S00-S09	Injuries to the head	S01<br>S02	Open wound of head<br>Fracture of skull and facial bones
S10-S19	Injuries to the neck	S13<br>S19	Dislocation and sprain of joints and ligaments at neck level<br>Other specified and unspecified injuries of neck
S20-S29	Injuries to the thorax	S20<br>S22	Superficial injury of thorax<br>Fracture of rib(s), sternum and thoracic spine
S30-S39	Injuries to the abdomen, lower back, lumbar spine, pelvis and external genitals	S30<br>S32	Superficial injury of abdomen, lower back, pelvis and external genitals<br>Fracture of lumbar spine and pelvis
S40-S49	Injuries to the shoulder and upper arm	S43<br>S46	Dislocation and sprain of joints and ligaments of shoulder girdle<br>Injury of muscle, fascia and tendon at shoulder and upper arm level
S50-S59	Injuries to the elbow and forearm	S52<br>S59	Fracture of forearm<br>Other and unspecified injuries of elbow and forearm
S60-S69	Injuries to the wrist, hand and fingers	S60<br>S63	Superficial injury of wrist, hand and fingers<br>Dislocation and sprain of joints and ligaments at wrist and hand level
S70-S79	Injuries to the hip and thigh	S72<br>S76	Fracture of femur<br>Injury of muscle, fascia and tendon at hip and thigh level
S80-S89	Injuries to the knee and lower leg	S82<br>S83	Fracture of lower leg, including ankle<br>Dislocation and sprain of joints and ligaments of knee
S90-S99	Injuries to the ankle and foot	S91<br>S99	Open wound of ankle, foot and toes<br>Other and unspecified injuries of ankle and foot
T07	Injuries involving multiple body regions	T07	Unspecified multiple injuries
T14	Injury of unspecified body region	T14	Injury of unspecified body region
T20-T25	Burns and corrosions of external body surface, specified by site	T22<br>T23	Burn and corrosion of shoulder and upper limb, except wrist and hand<br>Burn and corrosion of wrist and hand
T26-T28	Burns and corrosions confined to eye and internal organs	T27<br>T28	Burn and corrosion of respiratory tract<br>Burn and corrosion of other internal organs
T30-T32	Burns and corrosions of multiple and unspecified body regions	T30<br>T31	Burn and corrosion, body region unspecified<br>Burns classified according to extent of body surface involved

* Only abbreviated ICD-10-CM codes, containing the first three digits preceding the decimal point, are available in the public MEPS data. For brevity, several representative examples, but not all possible codes, are shown from each diagnosis code category.

© 2020 Liu et al. *JAMA Network Open.*

eTable 3. Diagnostic Groupings Used to Control for Changes in Injury Types Between Pre- and Post-ACA Periods

Individuals were assigned to one of the six diagnostic groups using CCS diagnosis codes for patients from 2010-2015 and ICD-10-CM diagnosis codes for patients from 2016-2017.

CCS Codes	CCS Code Description	ICD-10-CM Codes	ICD-10-CM Code Description	Unweighted Sample Size
205<br><br>226<br>227<br>228<br>230<br>233	Spondylosis; intervertebral disc disorders; other back problems<br>Fracture of neck of femur (hip)<br>Spinal cord injury<br>Skull and face fractures<br>Fracture of lower limb<br>Intracranial injury	M41<br>M43<br>M47<br>M50<br>M51<br>M53<br>M54<br>S02<br>S06<br>S09<br>S72<br>S82<br>S92	Scoliosis<br>Other deforming dorsopathies<br>Spondylosis<br>Cervical disc disorders<br>Thoracic, thoracolumbar, and lumbosacral intervertebral disc disorders<br>Other and unspecified dorsopathies, not elsewhere classified<br>Dorsalgia<br>Fracture of skull and facial bones<br>Intracranial injury<br>Other and unspecified injuries of head<br>Fracture of femur<br>Fracture of lower leg, including ankle<br>Fracture of foot and toe, except ankle	973
229<br>231	Fracture of upper limb<br>Other fractures	S22<br>S32<br>S42<br>S62	Fracture of rib(s), sternum and thoracic spine<br>Fracture of lumbar spine and pelvis<br>Fracture of shoulder and upper arm<br>Fracture at wrist and hand level	519
225<br><br>232	Joint disorders and dislocations; trauma-related<br>Sprains and strains	S13<br>S43<br>S46<br>S63<br>S76<br>S83<br>S86<br>S93	Dislocation and sprain of joints and ligaments at neck level<br>Dislocation and sprain of joints and ligaments of shoulder girdle<br>Injury of muscle, fascia and tendon at shoulder and upper arm level<br>Dislocation and sprain of joints and ligaments at wrist and hand level<br>Injury of muscle, fascia and tendon at hip and thigh level<br>Dislocation and sprain of joints and ligaments of knee<br>Injury of muscle, fascia and tendon at lower leg level<br>Dislocation and sprain of joints and ligaments at ankle, foot and toe level	1095

© 2020 Liu et al. *JAMA Network Open.*

234<br>235<br>236<br>239<br>240	Crushing injury or internal injury<br>Open wounds of head; neck; and trunk<br>Open wounds of extremities<br>Superficial injury; contusion<br>Burns	S00<br>S01<br>S20<br>S30<br>S40<br>S41<br>S60<br>S61<br>S70<br>S80<br>S81<br>S90<br>S91<br>T22<br>T23<br>T30	Superficial injury of head<br>Open wound of head<br>Superficial injury of thorax<br>Superficial injury of abdomen, lower back, pelvis and external genitals<br>Superficial injury of shoulder and upper arm<br>Open wound of shoulder and upper arm<br>Superficial injury of wrist, hand and fingers<br>Open wound of wrist, hand, and fingers<br>Superficial injury of hip and thigh<br>Superficial injury of knee and lower leg<br>Open wound of knee and lower leg<br>Superficial injury of ankle, foot and toes<br>Open wound of ankle, foot and toes<br>Burn and corrosion of shoulder and upper limb, except wrist and hand<br>Burn and corrosion of wrist and hand<br>Burn and corrosion, body region unspecified	1432
244	Other injuries and conditions due to external causes	S19<br>S39<br><br>S49<br>S59<br>S69<br>S79<br>S89<br>S99<br>T07<br>T14	Other specified and unspecified injuries of neck<br>Other and unspecified injuries of abdomen, lower back, pelvis and external genitals<br>Other and unspecified injuries of shoulder and upper arm<br>Other and unspecified injuries of elbow and forearm<br>Other and unspecified injuries of wrist, hand and finger(s)<br>Other and unspecified injuries of hip and thigh<br>Other and unspecified injuries of lower leg<br>Other and unspecified injuries of ankle and foot<br>Unspecified multiple injuries<br>Injury of unspecified body region	1277
Other / Missing	Various	Other / Missing	Various	992

© 2020 Liu et al. *JAMA Network Open.*

eTable 4. Sensitivity Analyses Using Alternate Regression Specifications for Out-of-Pocket Spending

Income Group (% FPL) and Model Specification	Out-of-Pocket Spending			
	**Pre-ACA Level (2017 $)**	**Adjusted Difference Post-ACA (%)**	**95% CI**	**p-value**
Full Sample				
(0) Main model	$1,105	-4.3	-15.5, +8.5	0.49
(1) Trauma patients identified by "INJURY" flag only	$1,122	-4.4	-16.7, +9.7	0.52
(2) Excluding patients ages 19-25	$1,215	-3.8	-15.9, +10.0	0.57
(3) Trauma patients with inpatient hospital stay only	$2,128	-18.8	-37.1, +4.9	0.11
Lowest (≤138)				
(0) Main model	$858	-30.4**	-46.6, -9.4	0.007
(1) Trauma patients identified by "INJURY" flag only	$855	-30.4*	-47.4, -7.9	0.011
(2) Excluding patients ages 19-25	$968	-34.9**	-51.2, -13.2	0.004
(3) Trauma patients with inpatient hospital stay only	$1,391	-28.0	-53.4, +11.2	0.14
Low (139-250)				
(0) Main model	$972	-21.4**	-34.5, -5.7	0.01
(1) Trauma patients identified by "INJURY" flag only	$973	-17.1*	-31.0, -0.4	0.046
(2) Excluding patients ages 19-25	$1,076	-18.5*	-32.2, -2.0	0.03
(3) Trauma patients with inpatient hospital stay only	$2,010	-55.7**	-69.5, -35.9	<0.001
Middle (251-400)				
(0) Main model	$1,346	-7.6	-28.3, +19.2	0.54
(1) Trauma patients identified by "INJURY" flag only	$1,399	-7.0	-28.8, +21.5	0.59
(2) Excluding patients ages 19-25	$1,445	+0.1	-23.7, +31.3	0.99
(3) Trauma patients with inpatient hospital stay only	$2,857	-5.5	-40.5, +50.2	0.81
High (>400)				
(0) Main model	$1,278	+14.1†	-2.1, +33.0	0.09
(1) Trauma patients identified by "INJURY" flag only	$1,277	+11.3	-5.3, +30.7	0.19
(2) Excluding patients ages 19-25	$1,368	+13.9	-2.7, +33.3	0.11
(3) Trauma patients with inpatient hospital stay only	$2,568	-9.9	-35.3, +25.6	0.54

†p<0.1, *p<0.05, **p<0.01

© 2020 Liu et al. *JAMA Network Open.*

eTable 5. Sensitivity Analyses Using Alternate Regression Specifications for Premium Spending

Income Group (% FPL) and Model Specification	Premium Spending			
	**Pre-ACA Level (2017 $)**	**Adjusted Difference Post-ACA (%)**	**95% CI**	**p-value**
Full Sample				
(0) Main model	$2,109	+8.0	-2.8, +20.0	0.15
(1) Trauma patients identified by "INJURY" flag only	$2,167	+5.9	-5.4, +18.5	0.32
(2) Excluding patients ages 19-25	$2,203	-2.4	-12.8, +9.1	0.66
(3) Trauma patients with inpatient hospital stay only	$2,131	-14.2	-36.6, +16.2	0.32
(4) MEPS definition of family	$2,156	+9.2†	-1.4, +21.0	0.09
Lowest (≤138)				
(0) Main model	$437	+1.2	-32.0, +50.6	0.95
(1) Trauma patients identified by "INJURY" flag only	$436	-2.5	-36.8, +50.4	0.91
(2) Excluding patients ages 19-25	$438	-7.5	-40.8, +44.8	0.73
(3) Trauma patients with inpatient hospital stay only	$338	-86.1†	-98.2, +8.0	0.06
(4) MEPS definition of family	$505	-2.9	-32.9, +40.6	0.88
Low (139-250)				
(0) Main model	$1,758	-20.4†	-37.2, +1.0	0.06
(1) Trauma patients identified by "INJURY" flag only	$1,735	-14.7	-33.0, +8.5	0.19
(2) Excluding patients ages 19-25	$1,840	-22.5*	-39.0, -1.4	0.04
(3) Trauma patients with inpatient hospital stay only	$1,963	-31.8	-64.7, +31.6	0.25
(4) MEPS definition of family	$1,816	-15.6	-32.8, +6.2	0.15
Middle (251-400)				
(0) Main model	$2,479	-0.5	-16.6, +18.7	0.95
(1) Trauma patients identified by "INJURY" flag only	$2,501	-0.4	-17.0, +19.5	0.97
(2) Excluding patients ages 19-25	$2,550	-9.4	-24.9, +9.2	0.30
(3) Trauma patients with inpatient hospital stay only	$2,783	-25.6	-54.1, +20.6	0.23
(4) MEPS definition of family	$2,501	+5.3	-11.7, +25.5	0.57
High (>400)				
(0) Main model	$3,743	+11.4†	-1.8, +26.4	0.09
(1) Trauma patients identified by "INJURY" flag only	$3,826	+7.0	-6.5, +22.5	0.32
(2) Excluding patients ages 19-25	$3,712	+6.7	-6.7, +22.0	0.34
(3) Trauma patients with inpatient hospital stay only	$4,095	-6.1	-32.6, +30.9	0.71
(4) MEPS definition of family	$3,779	+10.8	-2.2, +25.6	0.11

†p<0.1, *p<0.05, **p<0.01

© 2020 Liu et al. *JAMA Network Open.*

eTable 6. Sensitivity Analyses Using Alternate Regression Specifications for Out-of-Pocket and Premium Spending

Income Group (% FPL) and Model Specification	Out-of-Pocket + Premium Spending			
	Pre-ACA Level (2017 $)	Adjusted Difference Post-ACA (%)	95% CI	p-value
Full Sample				
(0) Main model	$3,953	-1.1	-8.7, +7.0	0.78
(1) Trauma patients identified by "INJURY" flag only	$4,043	-2.7	-10.6, +5.9	0.53
(2) Excluding patients ages 19-25	$4,117	-5.6	-13.0, +2.3	0.16
(3) Trauma patients with inpatient hospital stay only	$4,955	-15.8†	-31.3, +3.2	0.10
(4) MEPS definition of family	$4,048	-0.8	-8.1, +7.1	0.84
Lowest (≤138)				
(0) Main model	$1,624	-26.3**	-41.0, -8.1	0.006
(1) Trauma patients identified by "INJURY" flag only	$1,626	-28.5**	-43.8, -9.1	0.006
(2) Excluding patients ages 19-25	$1,645	-28.4*	-44.8, -7.1	0.01
(3) Trauma patients with inpatient hospital stay only	$1,904	-31.6†	-54.2, +2.0	0.06
(4) MEPS definition of family	$1,726	-24.1*	-38.9, -5.8	0.01
Low (139-250)				
(0) Main model	$3,332	-17.6*	-30.2, -2.8	0.02
(1) Trauma patients identified by "INJURY" flag only	$3,340	-13.9†	-27.0, +1.4	0.07
(2) Excluding patients ages 19-25	$3,561	-16.4*	-28.6, -2.2	0.03
(3) Trauma patients with inpatient hospital stay only	$4,846	-45.5**	-62.1, -21.7	0.001
(4) MEPS definition of family	$3,483	-15.4*	-27.9, -0.6	0.04
Middle (251-400)				
(0) Main model	$4,732	-3.4	-17.1, +12.6	0.66
(1) Trauma patients identified by "INJURY" flag only	$4,801	-3.1	-17.4, +13.6	0.70
(2) Excluding patients ages 19-25	$4,802	-5.6	-20.1, +11.6	0.50
(3) Trauma patients with inpatient hospital stay only	$6,559	-7.8	-38.4, +38.2	0.69
(4) MEPS definition of family	$4,773	-0.0	-13.7, +15.9	1.00
High (>400)				
(0) Main model	$6,146	+7.7	-1.7, +18.1	0.11
(1) Trauma patients identified by "INJURY" flag only	$6,227	+4.7	-5.1, +15.4	0.36
(2) Excluding patients ages 19-25	$6,133	+5.2	-4.2, +15.5	0.28
(3) Trauma patients with inpatient hospital stay only	$7,707	-8.3	-29.6, +19.4	0.52
(4) MEPS definition of family	$6,230	+6.4	-2.8, +16.4	0.18

†p<0.1, *p<0.05, **p<0.01

© 2020 Liu et al. *JAMA Network Open.*

eTable 7. Two-Part Model Results for Out-of-Pocket Spending (Sensitivity Analysis 6)

Stage 1: Likelihood of Any Out-of-Pocket Spending					
**Income Category (% of FPL)**	**Percent with Any OOP Spending Pre-ACA**	**Adjusted Odds Ratio Post-ACA**	**95% CI**		**p-value**
All patients	93.4	0.79	0.63	0.98	0.04*
Lowest (0-138)	88.6	0.74	0.53	1.04	0.09
Low (139-250)	92.9	0.67	0.40	1.10	0.11
Middle (251-400)	94.9	0.76	0.43	1.34	0.34
High (>400)	97.4	0.98	0.49	1.97	0.96
Stage 2: Changes in Mean OOP Spending Among Individuals with Nonzero OOP Spending					
**Income Category (% of FPL)**	**Mean Pre-ACA Spending Among Nonzero Spenders**	**Estimated Change in Spending, %**	**95% CI**		**p-value**
All patients	$1,183	-2.8	-14.2	+10.0	0.65
Lowest (0-138)	$969	-28.6	-44.8	-7.7	0.01*
Low (139-250)	$1,046	-18.4	-31.7	-2.6	0.03*
Middle (251-400)	$1,418	-6.4	-27.4	+20.7	0.61
High (>400)	$1,311	+13.6	-2.4	+32.2	0.10
Combined: Estimated Unconditional Changes in Mean Out-of-Pocket Spending					
**Income Category (% of FPL)**	**Mean Pre-ACA OOP Spending, 2017 Dollars**	**Estimated Change in Spending, 2017 Dollars**	**95% CI**		**p-value**
All patients	$1,105	-$44	-$177	+$89	0.52
Lowest (0-138)	$858	-$288	-$487	-$88	0.01**
Low (139-250)	$972	-$203	-$364	-$43	0.01*
Middle (251-400)	$1,346	-$99	-$425	+$228	0.55
High (>400)	$1,278	+$168	-$32	+$368	0.10

*p<0.05, **p<0.01
Note: Results shown here are from a two-part model with logit first stage (modeling the likelihood of having any nonzero spending) and GLM second stage with log link and gamma distribution (modeling the change in spending among individuals with nonzero spending).

© 2020 Liu et al. *JAMA Network Open.*

eTable 8. Two-Part Model Results for Premium Spending (Sensitivity Analysis 6)

Stage 1: Likelihood of Any Premium Spending					
**Income Category (% of FPL)**	**Percent with Any Premium Spending Pre-ACA**	**Adjusted Odds Ratio Post-ACA**	**95% CI**		**p-value**
All patients	55.0	0.94	0.81	1.09	0.40
Lowest (0-138)	14.7	1.07	0.73	1.55	0.73
Low (139-250)	51.1	0.76	0.54	1.05	0.09
Middle (251-400)	73.3	0.82	0.57	1.17	0.26
High (>400)	84.3	0.90	0.65	1.25	0.53
Stage 2: Changes in Mean Premium Spending Among Individuals with Nonzero Premium Spending					
**Income Category (% of FPL)**	**Mean Pre-ACA Spending Among Nonzero Spenders**	**Estimated Change in Spending, %**	**95% CI**		**p-value**
All patients	$3,837	+10.1	+1.4	+19.6	0.02*
Lowest (0-138)	$2,967	+8.8	-15.6	+40.1	0.51
Low (139-250)	$3,444	-3.7	-19.4	+15.1	0.68
Middle (251-400)	$3,381	+4.7	-8.8	+20.2	0.51
High (>400)	$4,439	+12.7	+0.9	+25.8	0.03*
Combined: Estimated Unconditional Changes in Mean Premium Spending					
**Income Category (% of FPL)**	**Mean Pre-ACA Premium Spending, 2017 Dollars**	**Estimated Change in Spending, 2017 Dollars**	**95% CI**		**p-value**
All patients	$2,109	+$162	-$40	+$363	0.12
Lowest (0-138)	$437	+$59	-$93	+$212	0.45
Low (139-250)	$1,758	-$257	-$594	+$81	0.14
Middle (251-400)	$2,479	-$21	-$441	+$399	0.92
High (>400)	$3,743	+$409	-$52	+$871	0.08

*p<0.05, **p<0.01

Note: Results shown here are from a two-part model with logit first stage (modeling the likelihood of having any nonzero spending) and GLM second stage with log link and gamma distribution (modeling the change in spending among individuals with nonzero spending).

© 2020 Liu et al. *JAMA Network Open.*

eTable 9. Two-Part Model Results for Out-of-Pocket and Premium Spending (Sensitivity Analysis 6)

Stage 1: Likelihood of Any Out-of-Pocket + Premium Spending					
**Income Category (% of FPL)**	**Percent with Any OOP + Premium Spending Pre-ACA**	**Adjusted Odds Ratio Post-ACA**	**95% CI**		**p-value**
All patients	97.1	0.72	0.52	1.01	0.05
Lowest (0-138)	93.9	0.63	0.43	0.92	0.02*
Low (139-250)	96.4	0.84	0.44	1.60	0.59
Middle (251-400)	99.3	0.61	0.17	2.15	0.44
High (>400)	99.5	1.55	0.13	17.79	0.72
Stage 2: Changes in Mean OOP + Premium Spending Among Individuals with Nonzero Spending					
**Income Category (% of FPL)**	**Mean Pre-ACA Spending Among Nonzero Spenders**	**Estimated Change in Spending, %**	**95% CI**		**p-value**
All patients	$4,069	-0.3%	-7.7%	+7.7%	0.94
Lowest (0-138)	$1,730	-23.4%	-38.7%	-4.4%	0.02*
Low (139-250)	$3,457	-18.0%	-30.2%	-3.6%	0.02*
Middle (251-400)	$4,767	-2.6%	-16.3%	+13.4%	0.74
High (>400)	$6,180	+7.7%	-2.4%	+18.7%	0.14
Combined: Estimated Unconditional Changes in Mean Out-of-Pocket + Premium Spending					
**Income Category (% of FPL)**	**Mean Pre-ACA OOP + Premium Spending, 2017 Dollars**	**Estimated Change in Spending, 2017 Dollars**	**95% CI**		**p-value**
All patients	$3,953	-$41	-$350	+$268	0.79
Lowest (0-138)	$1,624	-$432	-$753	-$111	0.01**
Low (139-250)	$3,332	-$623	-$1,123	-$123	0.02*
Middle (251-400)	$4,732	-$136	-$831	+$558	0.70
High (>400)	$6,146	+$476	-$146	+$1,099	0.13

*p<0.05, **p<0.01
Note: Results shown here are from a two-part model with logit first stage (modeling the likelihood of having any nonzero spending) and GLM second stage with log link and gamma distribution (modeling the change in spending among individuals with nonzero spending).

© 2020 Liu et al. *JAMA Network Open.*

eTable 10. Sensitivity Analyses Using Alternate Regression Specifications for Catastrophic Health Expenditures

**Income Group (% FPL) and Model Specification**	**Likelihood of CHE**			
	**Pre-ACA Level (%)**	**Adjusted OR / pp Change**	**95% CI**	**p-value**
Full Sample				
(0) Main model	12.2%	0.69**	0.54, 0.87	0.002
(1) Trauma patients identified by "INJURY" flag only	11.9%	0.67**	0.52, 0.86	0.002
(2) Excluding patients ages 19-25	11.9%	0.73*	0.58, 0.93	0.011
(3) Trauma patients with inpatient hospital stay only	20.7%	0.66	0.39, 1.12	0.12
(4) MEPS definition of family	11.5%	0.70**	0.55, 0.89	0.003
(5) Linear probability model (percentage points)	12.2%	-3.3**	-5.3, -1.3	0.001
Lowest (≤138)				
(0) Main model	28.9%	0.61**	0.44, 0.84	0.002
(1) Trauma patients identified by "INJURY" flag only	28.5%	0.55**	0.40, 0.77	<0.001
(2) Excluding patients ages 19-25	28.6%	0.63*	0.45, 0.90	0.011
(3) Trauma patients with inpatient hospital stay only	36.6%	0.68	0.38, 1.22	0.20
(4) MEPS definition of family	27.0%	0.59**	0.43, 0.82	0.002
(5) Linear probability model (percentage points)	28.9%	-8.4**	-13.7, -3.1	0.002
Low (139-250)				
(0) Main model	10.7%	0.72	0.43, 1.20	0.21
(1) Trauma patients identified by "INJURY" flag only	11.1%	0.72	0.43, 1.20	0.20
(2) Excluding patients ages 19-25	11.6%	0.78	0.46, 1.31	0.35
(3) Trauma patients with inpatient hospital stay only	26.9%	0.24*	0.07, 0.84	0.03
(4) MEPS definition of family	10.2%	0.74	0.44, 1.23	0.25
(5) Linear probability model (percentage points)	10.7%	-2.7	-6.9, +1.5	0.21
Middle (251-400)				
(0) Main model	5.0%	1.04	0.49, 2.22	0.91
(1) Trauma patients identified by "INJURY" flag only	5.1%	1.13	0.52, 2.45	0.76
(2) Excluding patients ages 19-25	5.6%	1.03	0.48, 2.18	0.94
(3) Trauma patients with inpatient hospital stay only	6.8%	1.95	0.49, 7.83	0.34
(4) MEPS definition of family	5.0%	1.18	0.56, 2.52	0.66
(5) Linear probability model (percentage points)	5.0%	+0.1	-3.4, +3.7	0.94
High (>400)				
(0) Main model	1.9%	0.83	0.35, 2.01	0.68
(1) Trauma patients identified by "INJURY" flag only	2.1%	0.76	0.32, 1.81	0.53
(2) Excluding patients ages 19-25	2.0%	0.79	0.33, 1.90	0.60
(3) Trauma patients with inpatient hospital stay only	6.3%	0.40	0.05, 3.01	0.37
(4) MEPS definition of family	1.9%	0.83	0.35, 2.01	0.68
(5) Linear probability model (percentage points)	1.9%	-0.5	-2.1, +1.2	0.59

†p<0.1, *p<0.05, **p<0.01; pp = percentage points

© 2020 Liu et al. *JAMA Network Open.*

eTable 11. Insurance Coverage Among Trauma Patients Aged 19 to 64 Years Who Experienced Catastrophic Health Expenditures in the Post-ACA Period (2014 to 2017)

Insurance Coverage*	%
Private	47.0
Marketplace	9.3
Medicaid	24.2
Uninsured	19.3

* Reflects insurance coverage sources in December of each study year. Individuals may report more than one source of insurance coverage, so percentages may sum to greater than 100%. “Private” includes commercial insurance coverage obtained through an employer, on the ACA marketplaces, or through other sources.

© 2020 Liu et al. *JAMA Network Open.*

eReferences.

1. Healthcare Cost and Utilization Project. CLINICAL CLASSIFICATIONS SOFTWARE (CCS) 2015. March 2016 [cited 2019 March 6]. Available from: https://www.hcup-us.ahrq.gov/toolssoftware/ccs/CCSUsersGuide.pdf.
2. Bureau of Labor Statistics. How BLS Measures Price Change for Medical Care Services in the Consumer Price Index. April 2019 [cited 2019 December 18]. Available from: https://www.bls.gov/cpi/factsheets/medical-care.htm.
3. Centers for Medicare and Medicaid Services. National Health Expenditure Data. December 2019 [cited 2019 December 18]. Available from: https://www.cms.gov/Research-Statistics-Data-and-Systems/Statistics-Trends-and-Reports/NationalHealthExpendData/index.

© 2020 Liu et al. *JAMA Network Open.*
